# Supplementary material for: Structure and distinct supramolecular organization of a PSII-ACPII dimer from a cryptophyte alga Chroomonas placoidea
Source: Nat Commun. 2024 May 28;15:4535. doi: 10.1038/s41467-024-48878-x (PMC11133340; doi:10.1038/s41467-024-48878-x)
Supplement: Supplementary file 1 — Supplementary Information [file 41467_2024_48878_MOESM1_ESM.pdf]

Supplementary Information for

Structure and distinct supramolecular organization of a PSII-ACPII dimer from a cryptophyte alga *Chroomonas placoidea*

Zhiyuan Mao, Xingyue Li, Zhenhua Li, Liangliang Shen, Xiaoyi Li, Yanyan Yang, Wenda Wang, Tingyun Kuang, Jian-Ren Shen, Guangye Han

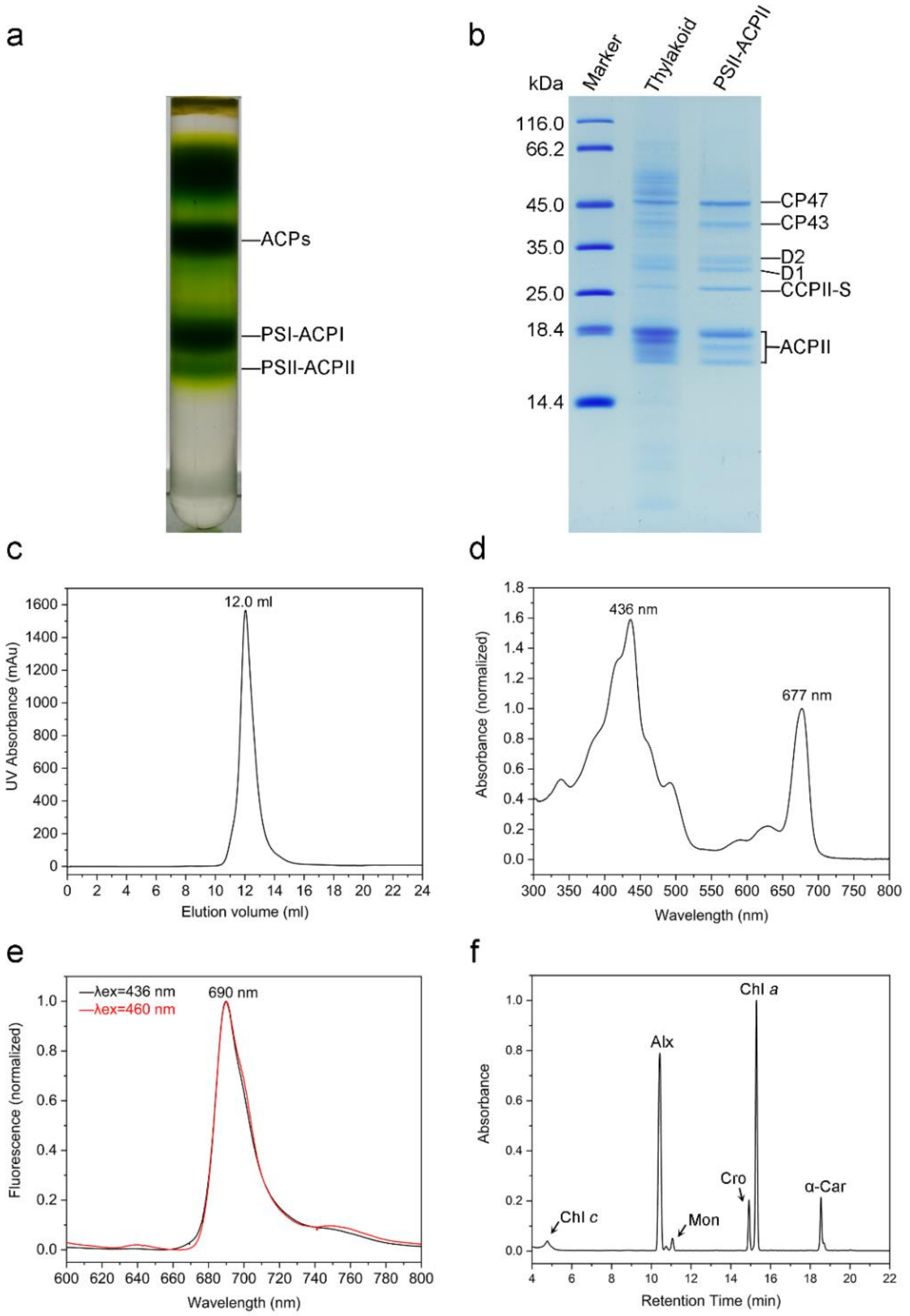

Supplementary Fig. 1. Sample preparation and characterization of the PSII-ACPII supercomplex from *C. placoidea*. **a**, Isolation of PSII-ACPII by sucrose density gradient (SDG) centrifugation. The fourth band labeled as PSII-ACPII was collected and used for single particle analysis in this study. **b**, SDS-PAGE analysis of the purified PSII-ACPII. The protein band CCPII-S was identified by mass spectrometry analysis. Lane 1: molecular weight marker (Thermo Scientific™: 26610); lane 2: thylakoid membranes (5 μg of Chl); lane 3: purified PSII-ACPII after size-exclusion chromatography (5 μg of Chl). **c**, Size-exclusion chromatography profile of the PSII-ACPII isolated by SDG with a Superose 6 Increase 10/300 GL column (Cytiva) at 4°C and monitored by absorption at 280 nm. **d**, Room-temperature absorption spectrum of PSII-ACPII. The spectrum was normalized to the maximum in the red region and showed high peaks at 436 nm and 677 nm. **e**, 77 K fluorescence emission spectra of PSII-ACPII excited at 436 and 460 nm, respectively. The spectra are normalized to the maximum emission peaks in the red region. **f**, Pigment analysis of PSII-ACPII by HPLC. Six major pigment peaks were identified as chlorophyll *c* (Chl *c*), alloxanthin (Alx), monadoxanthin (Mon), crocoxanthin (Cro), chlorophyll *a* (Chl *a*) and α-carotene (α-Car), based on the characteristic absorption spectrum of each peak fraction. Data in this figure is repeated more than three times, and all resulted in the same results. Source data are provided as a Source Data file.

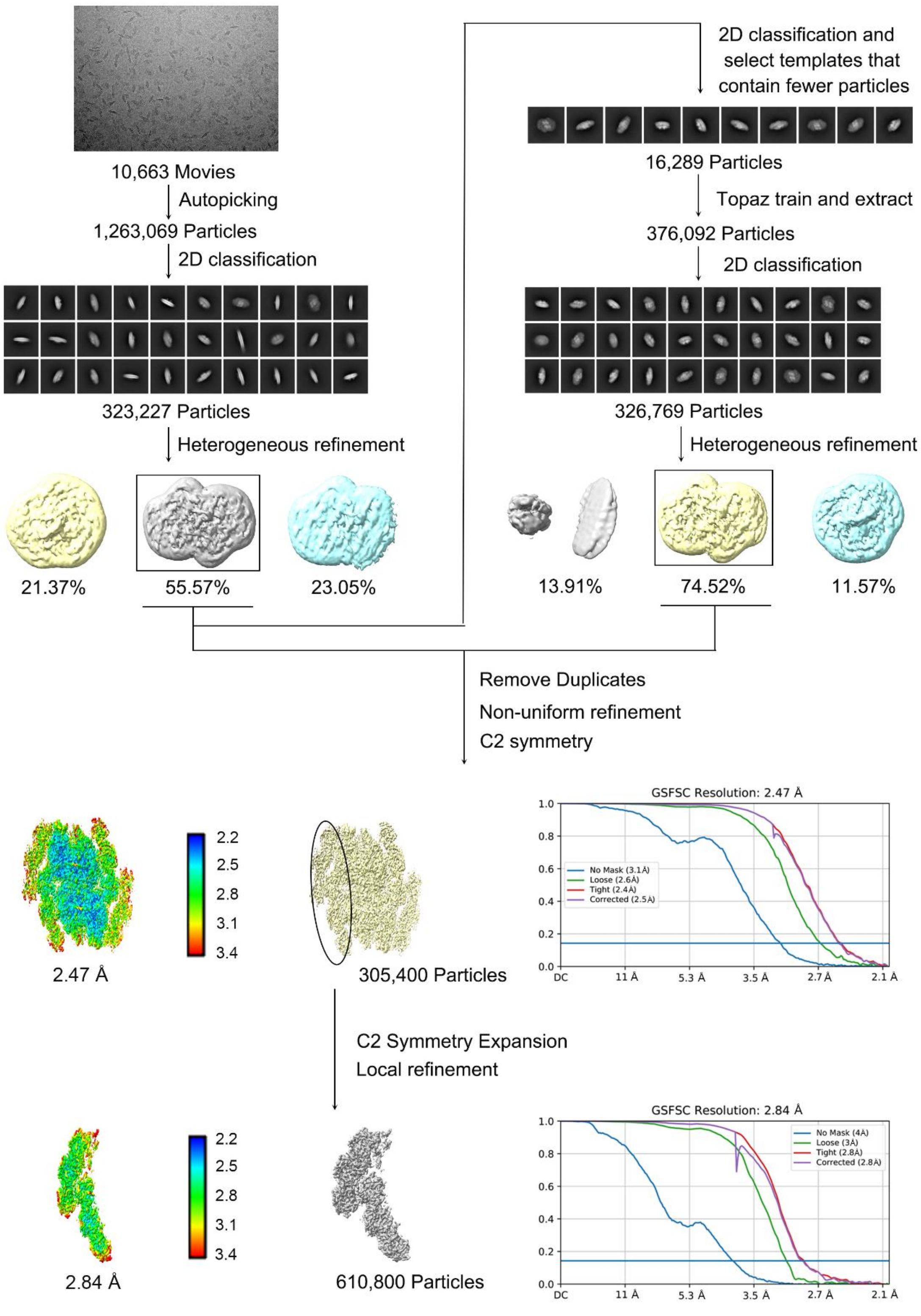

Supplementary Fig. 2. Single particle cryo-EM analysis of the PSII-ACPII supercomplex from *C. placoidea*. A typical cryo-EM image of PSII-ACPII and flow chart of the cryo-EM data processing. The global and local resolution maps of the PSII-ACPII were generated by cryoSPARC, in which moderately associated ACPs (ACPII-1-ACPII-6) were refined in a local map. The resolution (Å) of the global and local maps is colored by cryoSPARC. The gold standard FSC curves of the final 3D reconstruction are shown for the global and local maps, respectively.

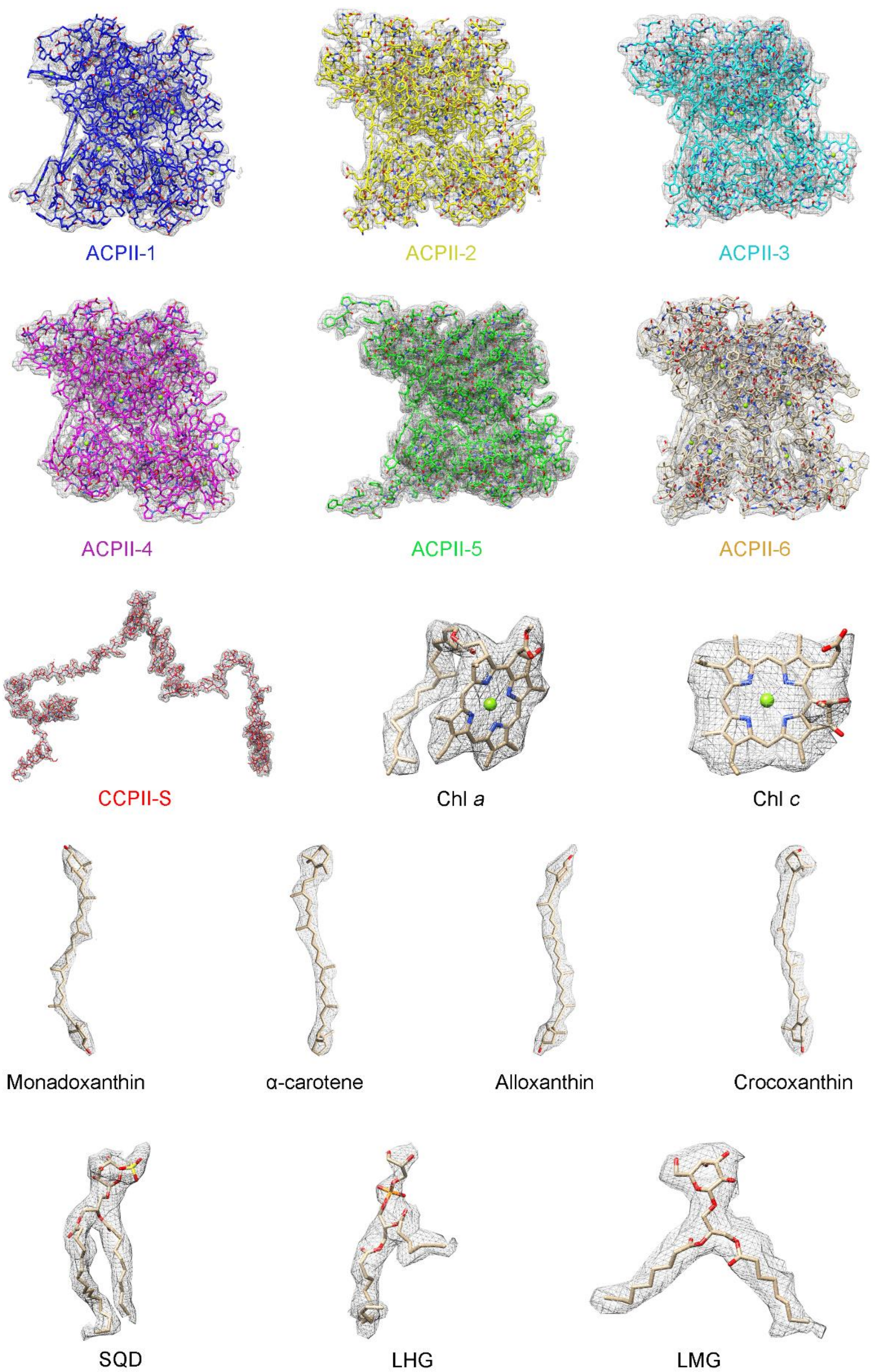

Supplementary Fig. 3. Density maps of ACPII subunits and cofactors in the PSII-ACPII supercomplex from *C. placoidea*. The ACPII subunits, pigments and lipid molecules in the PSII-ACPII supercomplex are displayed in a mixed cartoon/stick mode. The cryo-EM density maps of protein subunits and cofactors are depicted in gray meshes.

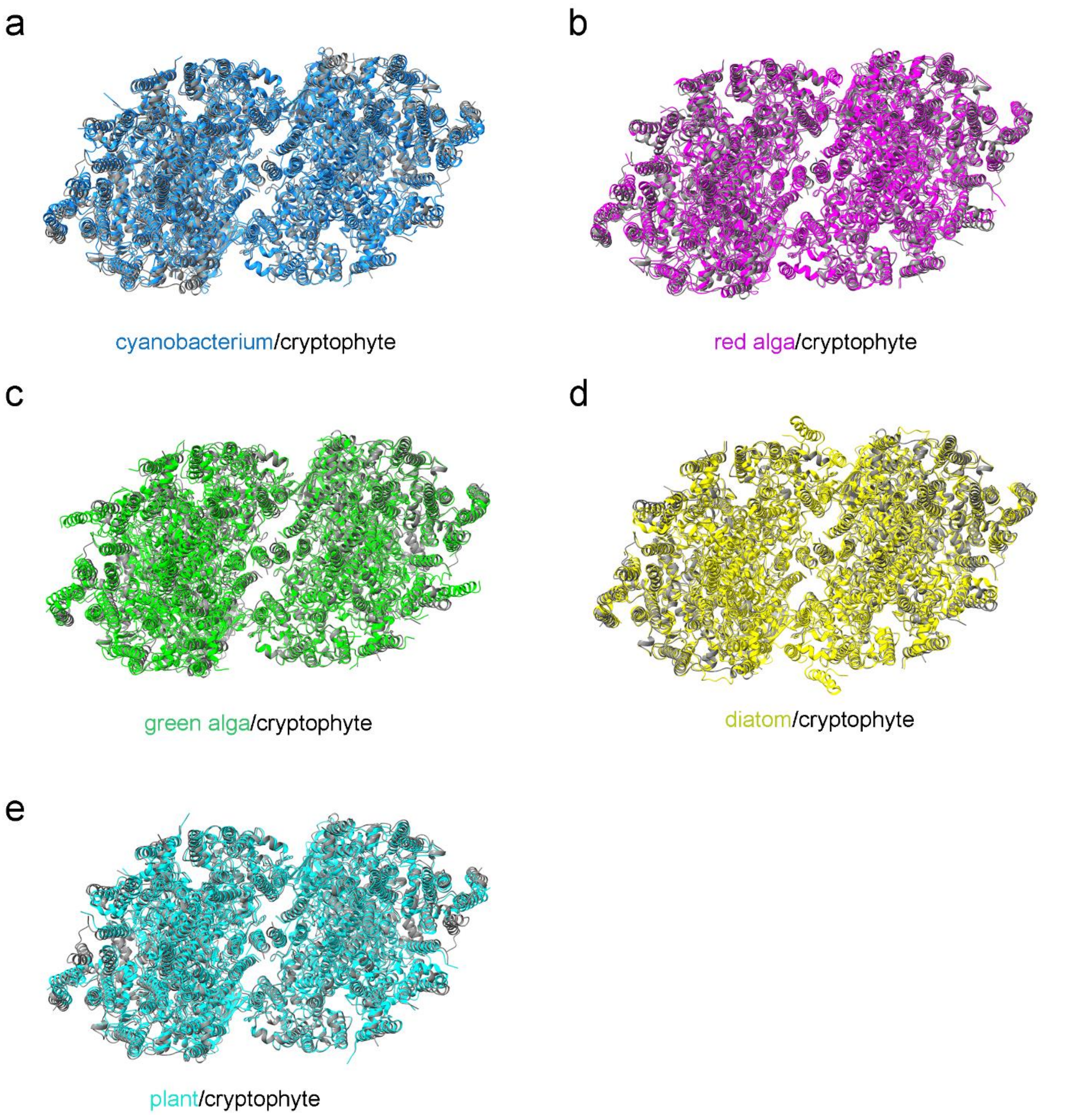

Supplementary Fig. 4. Structural comparison of the PSII core of the cryptophyte alga *C. placoidea* with that of other species. Superposition of the PSII core structures of the cryptophyte alga *C. placoidea* with that of a cyanobacterium (*T. vulcanus*) (PDB: 3WU2[https://doi.org/10.2210/pdb3wu2/pdb])[4] (**a**), a red alga (*C. caldarium*) (PDB: 4YUU[https://doi.org/10.2210/pdb4YUU/pdb])[34] (**b**), a green alga (*C. reinhardtii*) (PDB: 6KAC[https://doi.org/10.2210/pdb6KAC/pdb])[14] (**c**), a diatom (*C. gracilis*) (PDB: 7VD5[https://doi.org/10.2210/pdb7VD5/pdb])[16] (**d**), and a plant (*P. sativum*) (PDB: 5XNL[https://doi.org/10.2210/pdb5XNL/pdb])[18] (**e**). The PSII core structures are represented by cartoon modes, and the PSII core subunits of *C. placoidea*, *T. vulcanus, C. caldarium*, *C. reinhardtii*, *C. gracilis*, and *P. sativum* are colored in gray, blue, magenta, green, yellow, and cyan, respectively.

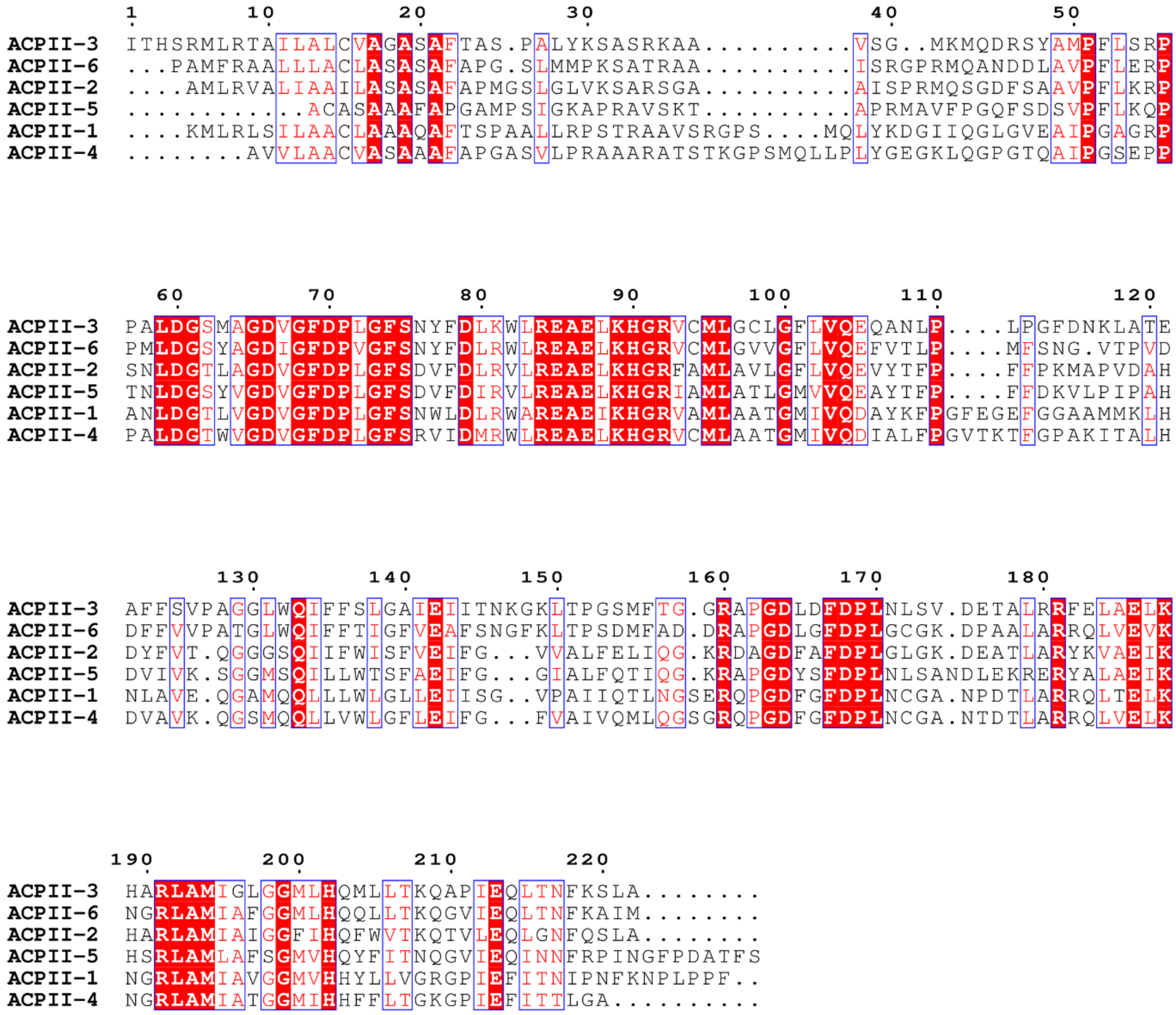

Supplementary Fig. 5. Sequence alignment of different ACPII subunits from *C. placoidea*. Fully conserved residues are shaded in red, and similar amino acids are highlighted by blue frames.

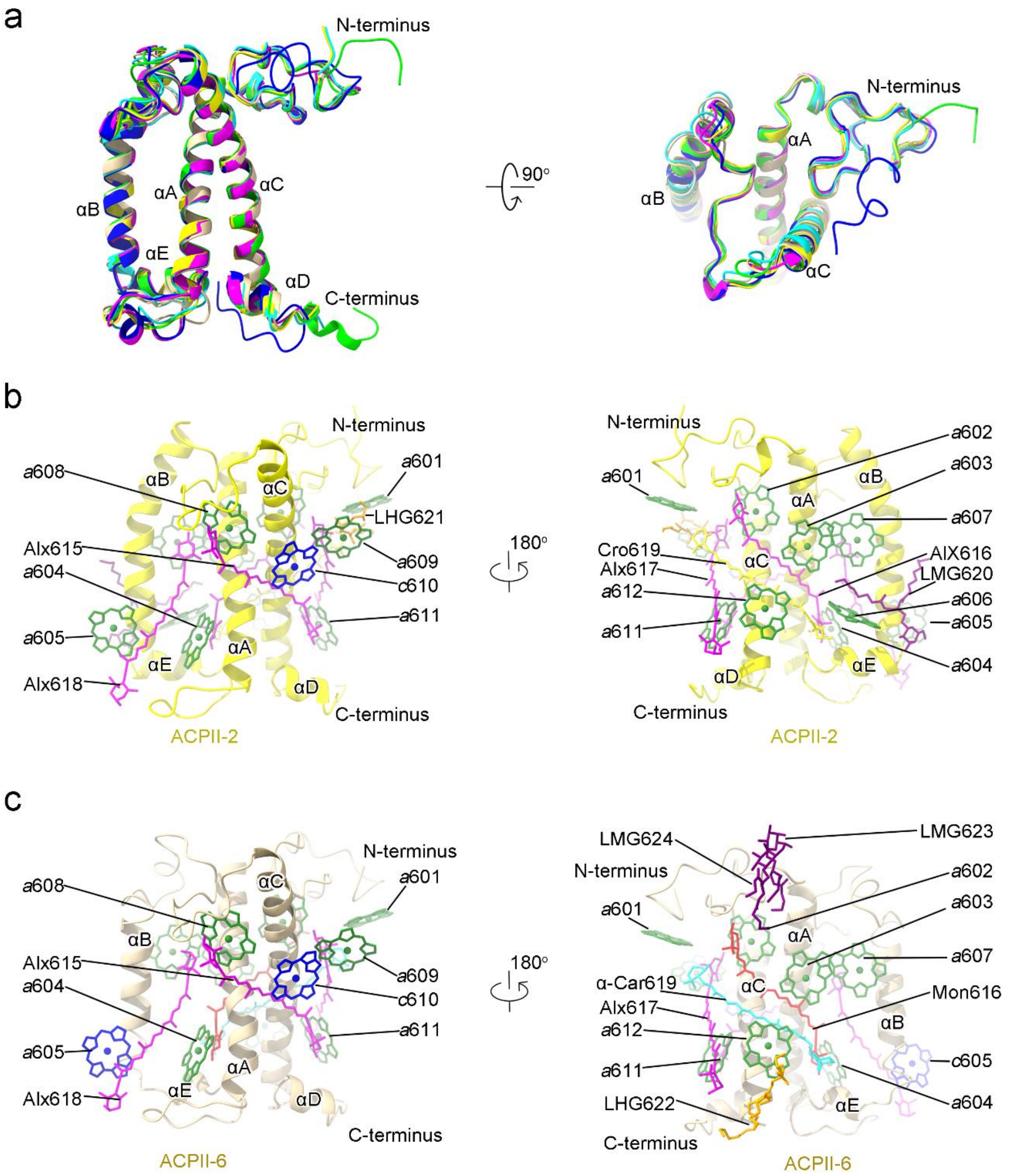

Supplementary Fig. 6. Structures of ACPII subunits from PSII-ACPII of *C. placoidea*. a, Structural comparison of ACPII-1/2/3/4/5/6 subunits. **b**, The structure of ACPII-2. **c**, The structure of ACPII-6. The apo-protein structures of ACPII subunits are depicted in transparent cartoon mode, whereas Chl *a*, Chl *c*, alloxanthin, α-carotene, monadoxanthin, crocoxanthin, LMG and LHG are depicted in sticks and colored as green, blue, magenta, cyan, red, yellow, purple and orange, respectively. For clarity, the phytol chain of the Chl molecules are omitted.

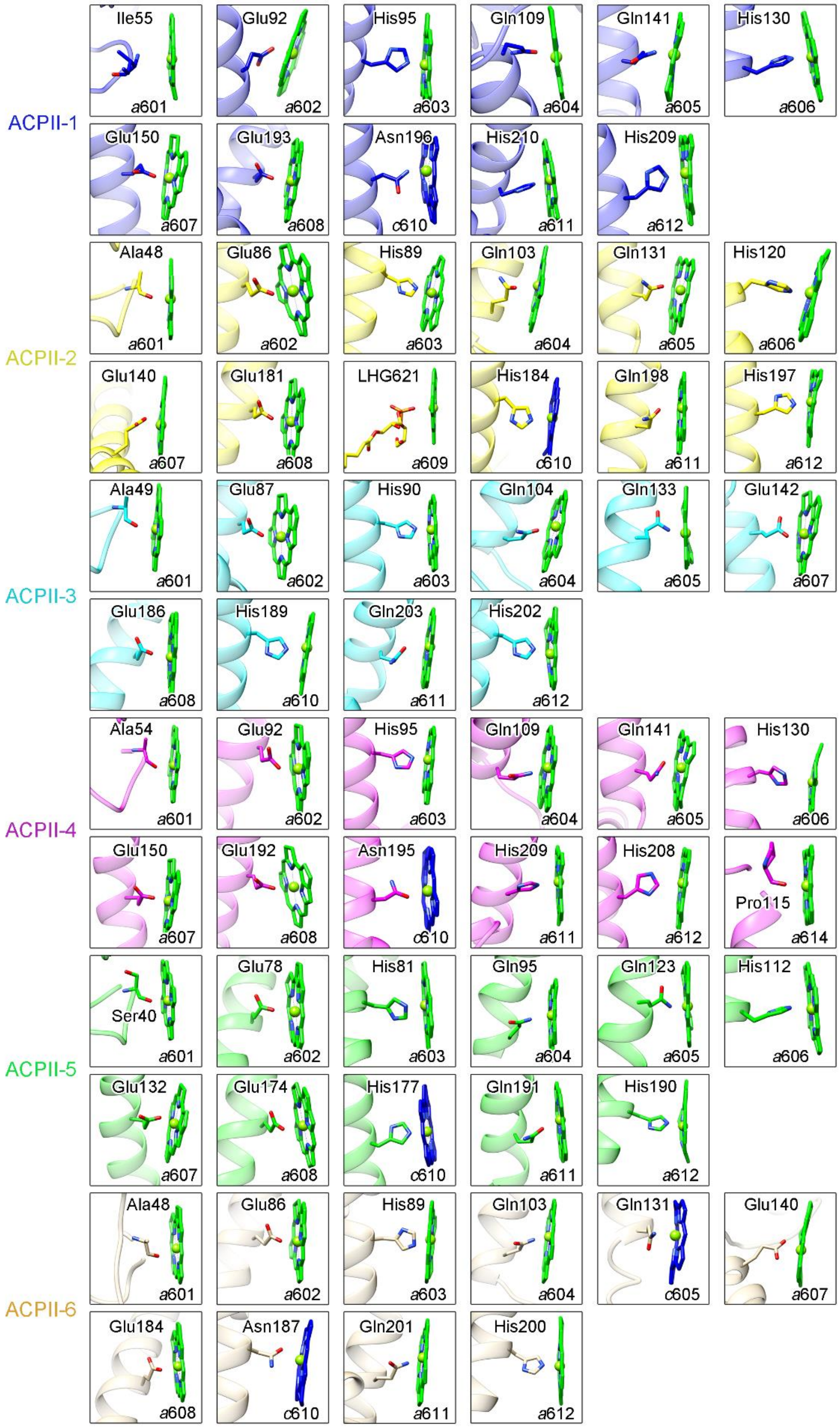

Supplementary Fig. 7. Interactions of Chls with their ligands in the ACPII subunits. Chl *a* and Chl *c* are colored in green and blue, respectively. For clarity, the phytol chain of the Chl molecules are omitted.

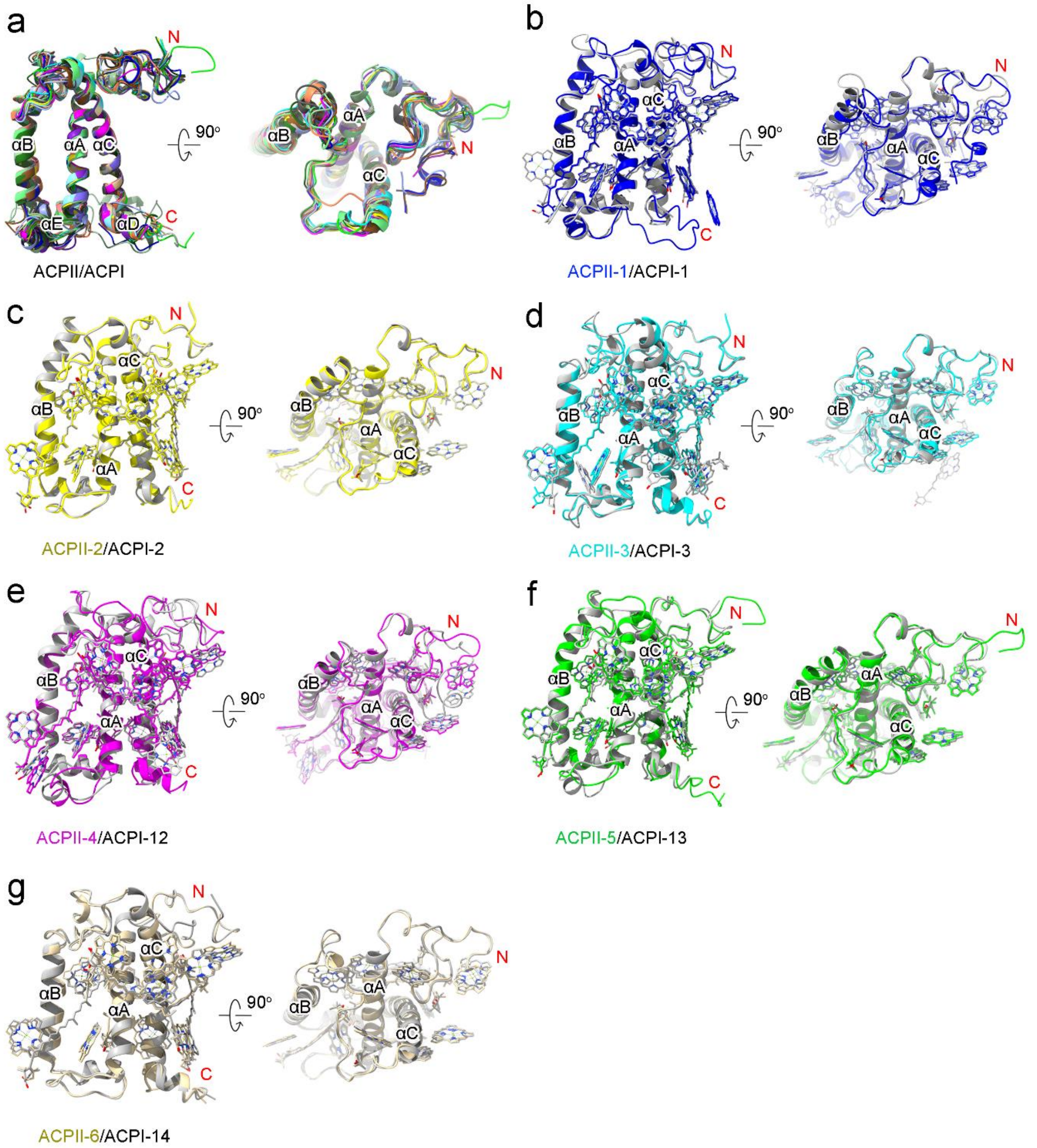

Supplementary Fig. 8. Structural comparisons of the individual ACPII and ACPI subunits of *C. placoidea*. a, Superposition of the structures of all 6 ACPIIs (ACPII-1/2/3/4/5/6) and 14 ACPIs (ACPI-1/2/3/4/5/6/7/8/9/10/11/12/13/14) observed in PSII-ACPII and PSI-ACPI (The PDB ID: 7Y7B[https://doi.org/10.2210/pdb7Y7B/pdb])[32]. **b-g,** Superposition of the structures between ACPII-1/ACPI-1 (**b**), ACPII-2/ACPI-2 (**c**), ACPII-3/ACPI-3 (**d**), ACPII-4/ACPI-12 (**e**), ACP-5/ACPI-13 (**f**), and ACPII-6/ACPI-14 (**g**). The apo-protein structures of ACPII and ACPI subunits are depicted in transparent cartoon mode, whereas the pigments are depicted in sticks and colored the same as that of the subunits, respectively. For clarity, the phytol chains of the Chl molecules are omitted.

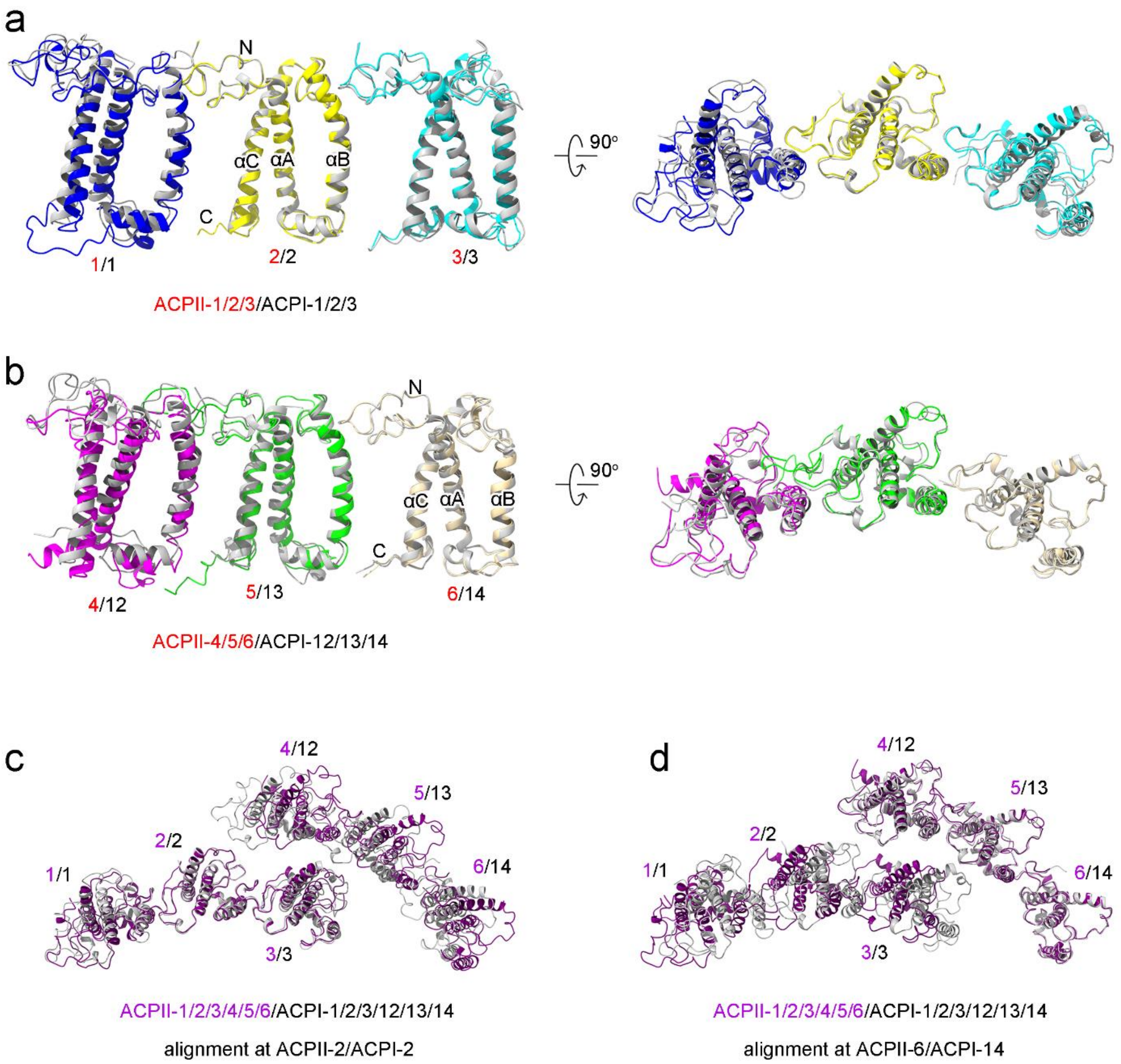

Supplementary Fig. 9. Structural comparison of the inner and outer ACPII belts with the corresponding ACPI belts in the two layers of the ACPIs module of *C. placoidea*. a, Superposition of the structures of ACPII-1/2/3 belt and ACPI-1/2/3 belt in PSI-ACPI (PDB ID: 7Y7B[https://doi.org/10.2210/pdb7Y7B/pdb])[32]. **b,** Superposition of the structures of ACPII-4/5/6 belt and ACPI-12/13/14 belt (PDB ID: 7Y7B[https://doi.org/10.2210/pdb7Y7B/pdb])[32]. **c**, **d**, Superposition of the overall structures of the inner and outer ACPII belts (purple) with the corresponding ACPI belts (gray) in the two layers of the ACPIs module of PSI-ACPI (PDB ID: 7Y7B[https://doi.org/10.2210/pdb7Y7B/pdb])[32] based on the ACPII-2/ACPI-2 subunits (**c**) and ACPII-6/ACPI-14 subunits (**d**), respectively. The apo-protein structures of ACPII and ACPI subunits are depicted in a transparent cartoon mode.

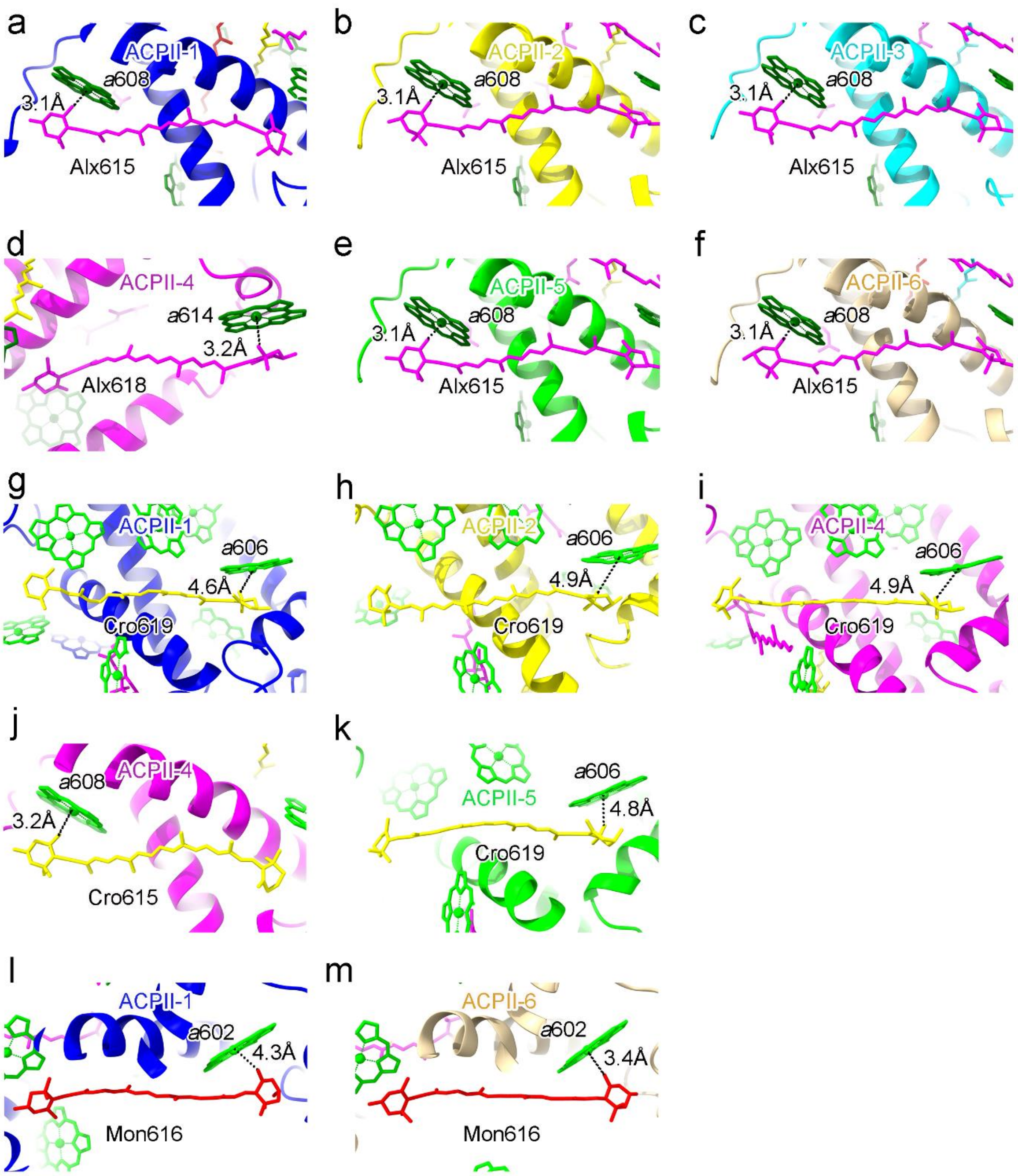

Supplementary Fig. 10. Binding environment of typical carotenoids (alloxanthin, crocoxanthin and monadoxanthin) in the ACPII subunits. a, Alx615 in ACPII-1. **b**, Alx615 in ACPII-2. **c,** Alx615 in ACPII-3. **d,** Alx618 in ACPII-4. **e,** Alx615 in ACPII-5. **f,** Alx615 in ACPII-6. **g,** Cro619 in ACPII-1. **h,** Cro619 in ACPII-2. **i,** Cro619 in ACPII-4. **j,** Cro615 in ACPII-4. **k,** Cro619 in ACPII-5. **l,** Mon616 in ACPII-1. **m,** Mon616 in ACPII-6. Chlorophylls, alloxanthins, crocoxanthin and monadoxanthin involved in the possible EETs or photoprotection are depicted in sticks and colored as green, magenta, yellow, and red, respectively. The shortest distances (Å) between carotenoids and Chls are labeled in black.

Supplementary Table 1. Cryo-EM data collection, structural analysis and refinement, and validation statistics.

Data collection and processing	PSII-ACPII (EMDB-37414) (PDB 8WB4)	ACPIIs (EMDB-38419) (PDB 8XKL)
Magnification	81,000 ×	81,000 ×
Voltage (kV)	300	300
Electron Exposure ($e^{-}/Å^2$)	60	60
Defocus range (μm)	-1.0 ~ -2.0	-1.0 ~ -2.0
Pixel size (Å)	1.04	1.04
Symmetry imposed	C2	C2
Number of initial particles	1,263,069	1,263,069
Number of final particles	305,400	610,800
Map resolution (Å)	2.47	2.84
FSC threshold	0.143	0.143
Refinement		
Number of atoms	80,246	15,357
Protein residues	62,272	10,211
Ligands	17,974	5,146
Root mean square deviations		
Bond lengths (Å)	0.004	0.005
Bond angles (°)	0.680	0.845
Validation		
Clashscore	7.06	7.22
Rotamer outliers (%)	0.08	0.39
Ramachandran plot		
Favored (%)	97.48	96.76
Allowed (%)	2.48	3.16
Disallowed (%)	0.04	0.08

Supplementary Table 2. Cofactors found in each subunit of the PSII-ACPII supercomplex.

Subunits	Traced residues	Chlorophylls	Carotenoids	Lipids	Others
**PsbA (D1)**	334 (10-343)	4 Chl *a*<br>2 Pheo	1 α-Car	2 SQD<br>1 LMG	1 Mn_4CaO_5<br>1 Fe ion, 1 Cl^-<br>1 plastoquinone
**PsbB (CP47)**	504 (2-505)	16 Chl *a*	3 α-Car	3 LMG, 1 DGD,<br>2 LHG, 1 SQD	
**PsbC (CP43)**	451 (11-461)	13 Chl *a*	3 α-Car	3 DGD, 1 LMG	
**PsbD (D2)**	342 (10-351)	2 Chl *a*	1 α-Car	3 LHG<br>1 LMG	1 plastoquinone<br>1 BCT
**PsbE**	77 (7-83)				
**PsbF**	30 (13-42)				1 haem
**PsbH**	65 (2-66)		1 α-Car	1 DGD, 1 LHG	
**PsbI**	35 (1-35)				
**PsbJ**	28 (6-33)				
**PsbK**	37 (9-45)		1 α-Car		
**PsbL**	38 (1-38)			1 LHG	
**PsbM**	36 (1-36)				
**PsbT**	32 (1-32)				
**PsbW**	48 (94-141)				
**PsbX**	39 (1-39)				
**PsbY**	34 (1-34)				
**PsbZ**	61 (1-61)				
**PsbO**	231 (69-324)				
**PsbU**	93 (58-150)				
**PsbV**	131 (33-163)				1 haem
**Unk1**	26 (1-26)				
**Unk2**	21 (1-21)				
**Unk3**	64 (1-64)	1 Chl *a*			
**ACPII-1**	194 (42-235)	12 Chl *a*<br>1 Chl *c*	3 Alx<br>1 Mon, 1 Cro		
**ACPII-2**	173 (44-216)	11 Chl *a*<br>1 Chl *c*	4 Alx<br>1 Cro	1 LMG<br>1 LHG	
**ACPII-3**	178 (45-222)	11 Chl *a*	1 α-Car, 4 Alx		
**ACPII-4**	172 (53-224)	12 Chl *a*<br>1 Chl *c*	3 Alx<br>2 Cro	1 LMG	
**ACPII-5**	187 (32-218)	11 Chl *a*<br>1 Chl *c*	4 Alx<br>1 Cro	1 SQD	
**ACPII-6**	173 (47-219)	9 Chl *a*<br>2 Chl *c*	1 α-Car<br>3 Alx<br>1 Mon	1 LMG<br>2 LHG	
**CCPII-S**	203 (83-285)	2 Chl *a*			
**Total**		112	40	28	8

Abbreviations used: **Chl *a***, chlorophyll *a*; **Chl *c***, chlorophyll *c*; **α-Car**, α-carotene; **Alx**, alloxanthin; **Mon**, monadoxanthin; **Cro**, crocoxanthin; **LMG**, edistearoylmonogalactosyl diglycerid; **SQD**, sulfoquinovosyldiacylglycerol; **LHG**, dipalmitoylphosphatidyl glycerol; **DGD,** digalactosyldiacyl glycerol; **BCT**, bicarbonate ion.

Supplementary Table 3. Pigment-binding sites in the ACPII subunits of PSII-ACPII.

	**ACPII-1**	**ACPII-2**	**ACPII-3**	**ACPII-4**	**ACPII-5**	**ACPII-6**	**CCPII-S**
601	Chl *a*	Chl *a*	Chl *a*	Chl *a*	Chl *a*	Chl *a*	Chl *a*
602	Chl *a*	Chl *a*	Chl *a*	Chl *a*	Chl *a*	Chl *a*	Chl *a*
603	Chl *a*	Chl *a*	Chl *a*	Chl *a*	Chl *a*	Chl *a*	
604	Chl *a*	Chl *a*	Chl *a*	Chl *a*	Chl *a*	Chl *a*	
605	Chl *a*	Chl *a*	Chl *a*	Chl *a*	Chl *a*	Chl *c*	
606	Chl *a*	Chl *a*		Chl *a*	Chl *a*		
607	Chl *a*	Chl *a*	Chl *a*	Chl *a*	Chl *a*	Chl *a*	
608	Chl *a*	Chl *a*	Chl *a*	Chl *a*	Chl *a*	Chl *a*	
609	Chl *a*	Chl *a*	Chl *a*	Chl *a*	Chl *a*	Chl *a*	
610	Chl *c*	Chl *c*	Chl *a*	Chl *c*	Chl *c*	Chl *c*	
611	Chl *a*	Chl *a*	Chl *a*	Chl *a*	Chl *a*	Chl *a*	
612	Chl *a*	Chl *a*	Chl *a*	Chl *a*	Chl *a*	Chl *a*	
613	Chl *a*						
614				Chl *a*			
615	Alx	Alx	Alx	Cro	Alx	Alx	
616	Mon	Alx	Alx	Alx	Alx	Mon	
617	Alx	Alx	Alx	Alx	Alx	Alx	
618	Alx	Alx	Alx	Alx	Alx	Alx	
619	Cro	Cro	α-Car	Cro	Cro	α-Car	
620		LMG		LMG			
621		LHG					
622						LHG	
623						LMG	
624						LMG	
625					SQD		

Abbreviations used: **Chl *a***, chlorophyll *a*; **Chl *c***, chlorophyll *c;* **Alx**, alloxanthin; **Mon**, monadoxanthin; **α-Car**, α-carotene; **Cro**, crocoxanthin; **LMG**, edistearoylmonogalactosyl diglyceride; **SQD**, sulfoquinovosyldiacylglycerol; **LHG**, dipalmitoylphosphatidyl glycerol.

Supplementary Table 4. Calculated FRET rates in the PSII-ACPII supercomplex.

Pigments	FRET rate, k_{FRET} (ps^{-1})	Lifetime, τ (ps)	Half-life, $t_{1/2}$ (ps)	Distance, R(Å)	Dipole orientation factor, K^2
$a608_{ACPII-1}$—$a601_{ACPII-2}$	0.775	1.291	0.895	16.722	3.031
$a608_{ACPII-2}$—$a601_{ACPII-3}$	0.792	1.262	0.875	16.609	2.976
$a608_{ACPII-4}$—$a601_{ACPII-5}$	0.589	1.696	1.176	17.455	2.983
$a608_{ACPII-5}$—$a601_{ACPII-6}$	0.765	1.307	0.906	16.677	2.946
$a605_{ACPII-1}$—$a611_{ACPII-2}$	0.962	1.039	0.720	8.331	0.058
$a605_{ACPII-1}$—$a612_{ACPII-2}$	0.509	1.962	1.360	14.895	0.996
$a605_{ACPII-2}$—$a611_{ACPII-3}$	1.321	0.757	0.525	8.323	0.079
$a605_{ACPII-2}$—$a612_{ACPII-3}$	0.383	2.614	1.812	14.922	0.756
$a605_{ACPII-4}$—$a611_{ACPII-5}$	0.893	1.119	0.776	8.426	0.057
$a605_{ACPII-4}$—$a612_{ACPII-5}$	0.212	4.711	3.265	15.467	0.520
$a605_{ACPII-5}$—$a611_{ACPII-6}$	0.387	2.586	1.792	8.217	0.021
$a605_{ACPII-5}$—$a612_{ACPII-6}$	0.415	2.407	1.668	14.962	0.833
$a606_{ACPII-5}$—$a601_{CCPII-S}$	0.350	2.857	1.980	15.965	1.037
$a601_{CCPII-S}$—$a612_{ACPII-6}$	0.835	1.198	0.830	10.583	0.210
$a603_{ACPII-4}$—$a610_{ACPII-3}$	0.163	6.149	4.262	13.701	0.193
$a603_{ACPII-4}$—$a609_{ACPII-3}$	0.603	1.658	1.149	13.963	0.799
$a607_{ACPII-4}$—$a610_{ACPII-3}$	0.734	1.362	0.944	13.736	0.882
$a606_{ACPII-4}$—$a611_{ACPII-3}$	0.015	66.585	46.153	24.798	0.625
$a606_{ACPII-4}$—$a604_{ACPII-3}$	0.025	39.852	27.623	26.127	1.428
$a612_{ACPII-5}$—$a604_{ACPII-3}$	0.011	91.139	63.173	22.629	0.264
$a606_{ACPII-5}$—$a605_{ACPII-3}$	0.013	76.036	52.704	23.404	0.387
$a607_{ACPII-2}$—$a602_{CCPII-S}$	0.221	4.516	3.131	17.178	1.018
$a603_{ACPII-2}$—$a602_{CCPII-S}$	0.177	5.641	3.910	21.555	3.181
$a602_{CCPII-S}$—$a615_{CP47}$	1.291	0.775	0.537	15.811	3.608
$a603_{ACPII-6}$—$a101_{Unk3}$	1.083	0.924	0.640	12.655	0.796
$a607_{ACPII-6}$—$a101_{Unk3}$	0.421	2.376	1.647	13.342	0.425
$a602_{ACPII-6}$—$a101_{Unk3}$	0.185	5.402	3.744	20.484	2.447
$a101_{Unk3}$—$a507_{CP43}$	0.152	6.589	4.568	21.027	2.346
$a101_{Unk3}$—$a513_{CP43}$	0.419	2.386	1.654	15.841	1.185
$a607_{ACPII-3}$—$a506_{CP43}$	0.049	20.496	14.207	26.379	2.941
$a606_{ACPII-1}$—$a601_{CP47}$	0.099	10.121	7.015	20.090	1.163
$a612_{ACPII-2}$—$a601_{CP47}$	0.041	24.668	17.099	17.926	0.241

The lifetime (τ) and half-life ($t_{1/2}$) were defined as $\tau = 1/k_{FRET}$ and $t_{1/2} = 0.693/k_{FRET}$, respectively.
