# Supplementary material for: Structure and distinct supramolecular organization of a PSII-ACPII dimer from a cryptophyte alga Chroomonas placoidea
Source: Nat Commun. 2024 May 28;15:4535. doi: 10.1038/s41467-024-48878-x (PMC11133340; doi:10.1038/s41467-024-48878-x)
Supplement: Supplementary file 3 — Reporting Summary [file 41467_2024_48878_MOESM3_ESM.pdf]

nature portfolio

Corresponding author(s): Guangye Han

Last updated by author(s): Apr 12, 2024

Reporting Summary

Nature Portfolio wishes to improve the reproducibility of the work that we publish. This form provides structure for consistency and transparency in reporting. For further information on Nature Portfolio policies, see our Editorial Policies and the Editorial Policy Checklist.

Statistics

For all statistical analyses, confirm that the following items are present in the figure legend, table legend, main text, or Methods section.

n/a	Confirmed	
☐	☒	The exact sample size (*n*) for each experimental group/condition, given as a discrete number and unit of measurement
☐	☒	A statement on whether measurements were taken from distinct samples or whether the same sample was measured repeatedly
☒	☐	The statistical test(s) used AND whether they are one- or two-sided<br>*Only common tests should be described solely by name; describe more complex techniques in the Methods section.*
☒	☐	A description of all covariates tested
☒	☐	A description of any assumptions or corrections, such as tests of normality and adjustment for multiple comparisons
☒	☐	A full description of the statistical parameters including central tendency (e.g. means) or other basic estimates (e.g. regression coefficient) AND variation (e.g. standard deviation) or associated estimates of uncertainty (e.g. confidence intervals)
☒	☐	For null hypothesis testing, the test statistic (e.g. *F*, *t*, *r*) with confidence intervals, effect sizes, degrees of freedom and *P* value noted<br>*Give P values as exact values whenever suitable.*
☒	☐	For Bayesian analysis, information on the choice of priors and Markov chain Monte Carlo settings
☒	☐	For hierarchical and complex designs, identification of the appropriate level for tests and full reporting of outcomes
☒	☐	Estimates of effect sizes (e.g. Cohen's *d*, Pearson's *r*), indicating how they were calculated

Our web collection on statistics for biologists contains articles on many of the points above.

Software and code

Policy information about availability of computer code

Data collection	EPU Version 3.0.0.4164REL
Data analysis	cryoSparc v4.2.1, Coot 0.9.7, Pymol 2.6.0a0, Chimera 1.16, ChimeraX 1.4, Phenix 1.20.1, Topaz 0.2.4

For manuscripts utilizing custom algorithms or software that are central to the research but not yet described in published literature, software must be made available to editors and reviewers. We strongly encourage code deposition in a community repository (e.g. GitHub). See the Nature Portfolio guidelines for submitting code & software for further information.

Data

Policy information about availability of data

All manuscripts must include a data availability statement. This statement should provide the following information, where applicable:

- Accession codes, unique identifiers, or web links for publicly available datasets
- A description of any restrictions on data availability
- For clinical datasets or third party data, please ensure that the statement adheres to our policy

The cryo-EM density map and atomic model for the PSII-ACPII supercomplex structure have been deposited in the Electron Microscopy Data Bank and the Protein Data Bank (EMD ID code 37414 and PDB ID code 8WB4), and the locally refined cryo-EM map and atomic model of the ACPIIs have been deposited in the Electron Microscopy Data Bank and the Protein Data Bank (EMD-38419 and 8XKL). The RNA-seq data have been deposited in in the NCBI Sequence Read Archive (SRA)

database (accession code PRJNA1095808). The data that support the findings of this study are available from the corresponding authors upon request. Other data are cited from the previously published papers and PDB data bank, and they are clearly indicated in the text where appropriate.

Research involving human participants, their data, or biological material

Policy information about studies with human participants or human data. See also policy information about sex, gender (identity/presentation), and sexual orientation and race, ethnicity and racism.

Reporting on sex and gender	N/a
Reporting on race, ethnicity, or other socially relevant groupings	N/a
Population characteristics	N/a
Recruitment	N/a
Ethics oversight	N/a

Note that full information on the approval of the study protocol must also be provided in the manuscript.

Field-specific reporting

Please select the one below that is the best fit for your research. If you are not sure, read the appropriate sections before making your selection.

☒ Life sciences ☐ Behavioural & social sciences ☐ Ecological, evolutionary & environmental sciences

For a reference copy of the document with all sections, see nature.com/documents/nr-reporting-summary-flat.pdf

Life sciences study design

All studies must disclose on these points even when the disclosure is negative.

Sample size	No statistic method was used to predetermine the sample size. The number of cryo-EM micrographs collected was based on the previous knowledge and the results indicated that the size is sufficient to generate a density map at an atomic resolution for PSII-ACPII supercomplex.
Data exclusions	No data exclusion criteria was pre-established. 2D and 3D classification were used to obtain multiple classes. Only the particles in the classes that showed clear structural signals and intact structures were chosen, combined and used in the final reconstruction of cryo-EM map and refinement. Details are described in the flowchart of Supplementary Fig. 2. and Methods.
Replication	Multiple rounds of structural refinement have been performed and all resulted in same density maps, although at different resolutions. All of experiments concerning the purification and characterization of PSII-ACPII ((detergent-solubilization, sucrose density gradient centrifugation, SDS-PAGE, absorption spectrum, fluorescence emission spectra and pigments analysis) have been repeated independently for more than 3 times, and all showed the similar results.
Randomization	Randomization of samples is not relevant to our study to solve the structure of a specific protein supercomplex, because no grouping is needed and the results are not subjective. For single-particle cryo-electron microscopy analysis, in which the dataset is divided into two random sets for reconstruction, refinement and Gold standard Fourier Shell Correlation method was used to estimate the resolution of the cryo-EM structures.
Blinding	All experiments are not blinded. Blinding is not relevant to our study because we are studying the structure of a specific protein supercomplex.

Reporting for specific materials, systems and methods

We require information from authors about some types of materials, experimental systems and methods used in many studies. Here, indicate whether each material, system or method listed is relevant to your study. If you are not sure if a list item applies to your research, read the appropriate section before selecting a response.

Materials & experimental systems

n/a	Involved in the study
☒	☐ Antibodies
☒	☐ Eukaryotic cell lines
☒	☐ Palaeontology and archaeology
☒	☐ Animals and other organisms
☒	☐ Clinical data
☒	☐ Dual use research of concern
☒	☐ Plants

Methods

n/a	Involved in the study
☒	☐ ChIP-seq
☒	☐ Flow cytometry
☒	☐ MRI-based neuroimaging

Plants

Seed stocks	N/a
Novel plant genotypes	N/a
Authentication	N/a
